# Demonstration of Interposed Modular Hydrogel Sheet for Multicellular Analysis in a Microfluidic Assembly Platform

**DOI:** 10.1038/s41598-017-01363-6

**Published:** 2017-05-02

**Authors:** Chae Yun Bae, Jaejung Son, Hail Kim, Je-Kyun Park

**Affiliations:** 1 Department of Bio and Brain Engineering, Korea Advanced Institute of Science and Technology (KAIST), Daejeon, 34141 Republic of Korea; 2 Graduate School of Medical Science and Engineering, Korea Advanced Institute of Science and Technology (KAIST), Daejeon, 34141 Republic of Korea

## Abstract

Hydrogel sheets have emerged as a promising biomaterial scaffold for the encapsulation and transfer of multicellular structures. Although the improvement of the chemical interactions and the design of micro-scaled geometry have contributed to the development of multipurpose hydrogel scaffolds, the application of hydrogel sheets to assess multicellular structures is still challenging. To expand the technical applicability of hydrogel sheets, we here demonstrate that a single layer of the hydrogel sheet can be integrated as an interposed module in a microfluidic device for multicellular analysis. As a cell culture unit, encapsulated pancreatic insulinoma (MIN6) cells in the hydrogel sheet were labeled and examined via multiple microchannels. After obtaining simultaneously multi-labeled cells in the hydrogel sheet that had been incorporated into the microfluidic device, each modular hydrogel sheet was also recoverable and re-cultured without any distortion. The modular hydrogel sheet can be simply manipulated and conserved as a multicellular module in a three-dimensional (3D) *in vitro* culture platform. Using the modular concept of hydrogel sheets capable of cell culture and/or assay, an integrated multicellular analysis in the microfluidic device is expected to improve accessibility, scalability, and practicality for end users.

## Introduction

Microfluidic techniques have been extensively explored with the ability to analyze multicellular structures (e.g., spheroids, islets, embryoid body, or any shape of functional three-dimensional (3D) multicellular structure) to study typical morphogenesis^1–8^, derive differentiation^3, 9^, assess viability against drugs^2, 10, 11^, analyze their physiological functions^2, 5, 7, 12–14^, and circulate nutrients via a co-culture system^15^ under the shear-inducing dynamic flows^1, 6, 12, 15–18^ or regulated gradients of the metabolites^1, 13, 19^. In order to complete this type of analysis under microfluidic devices, especially in spheroids, several fabrication methods, such as generating droplets^10^, trapping on microwells^2, 11^, hanging drops^15^ and bioprinting^8^, were carried out for the mass-produced multicellular structures. Then, an additional process for effective production, individual capturing, and high-density rearrangement of these multicellular structures in this microfluidic device was inevitable for rapid, multiple analysis. Most approaches using microfluidics favor a rapidly reactive, multiplexed analysis tool; however, the multicellular structures have thus far only been analyzed in a few ways, such as with capturing confirmed multicellular structures in the microfluidic devices^2, 15, 16, 18^, introducing empty scaffolds to generate multicellular structures within the devices^1, 7^, and exploiting patterned scaffolds on bare surfaces as a multiple array^9–11, 19^. According to previous attempts to analyze multicellular structures, there were some drawbacks and limitations: low efficiency in capturing the particle-sized multicellular constructs in a flow condition, an additional procedure for localizing empty scaffolds and culturing multicellular structures within the devices, and limited space and a lack of proper microenvironment to manipulate massive multicellular structures in the microfluidic device. Accordingly, since pretreatment processes have become more complicated for the microfluidic devices, both user accessibility and technical practicality have been dramatically hindered and reduced. Thus, an improved multicellular analysis under the shear-inducing dynamic flow within microfluidic devices, where both hydrogels and cells can be simply manipulated, is still necessary to improve technical accessibility.

Hydrogel sheets, as carriers of multicellular structures, can address the shortcomings in the effective rearrangement of cells per macro-scale unit area by utilization of different types of cells or hydrogel biomaterials (e.g., collagen^20^, alginate^21^, poly(N-isopropylacrylamide)-coated surface)^22^. These macro-scale sheet-like multicellular structures can provide (1) a simple and effective tool for the reorganization of cells due to micropatterned substrates^20, 21, 23^, (2) a large contact surface for simultaneous screening based on multiple arrayed cells, (3) an advanced artificial tissue layer by utilizing various cell types^20^, and (4) a novel application for large tissue-like multi-layered sheets^22, 24, 25^. Thus, hydrogel sheets can be applied as a tool to analyze multicellular structures integrated with microfluidic devices that require additional processes for individual rearrangement. However, these multicellular hydrogel sheets have rarely been applied in microfluidic devices for multicellular analysis under the shear-inducing dynamic flow conditions. To efficiently perform microfluidic cell-based analysis using hydrogel sheets, each component should be separately manipulated and selected to play functional roles such as long-term *in vitro* culture and multiple assay assessment.

Here, we focused on the demonstration of an integration method between microfluidic channels and a sheet of hydrogel construct, as a hydrogel-incorporating microfluidic assembly platform, to show the possibility of a general-purpose cell-based assay platform. To determine cell viability, toxicity, proliferation and immunoassay in this system, pancreatic insulinoma (MIN6) cells were cultured in the hydrogel sheet and demonstrated for the assay in the microfluidic assembly platform. For the *in vitro* assay, MIN6 cells can be an adequate cell type replacing pancreatic beta cells that are limited to isolate from animal sources and no longer remain in their primary state of viability and function^26, 27^. In our previous work^23^, it was reported that MIN6 cells were clumped together as a multicellular cluster in a micropatterned free-standing hydrogel sheet; moreover, there was a functional improvement with inclusion of mesh micropatterns in the hydrogel sheet. On the basis of the advantages of hydrogel sheets as a cell-carrier module, here we reversibly integrated a layer of hydrogel sheet as an insertional module into a microfluidic device, with the aim to demonstrate the potential application of the hydrogel module-based multicellular analysis using MIN6 cell clusters. In addition, we present an optimal process to incorporate the micropatterned hydrogel sheet into a microfluidic device and complete it with multicellular clusters^23^. This allows us to expand the application of 3D modular sheets for *in vitro* cell culture and analysis and suggests a universally operated microfluidic interface based on independent hydrogel sheets.

## Results

### Assembly process of the modular hydrogel sheet with the PDMS chip

**Figure 1:**
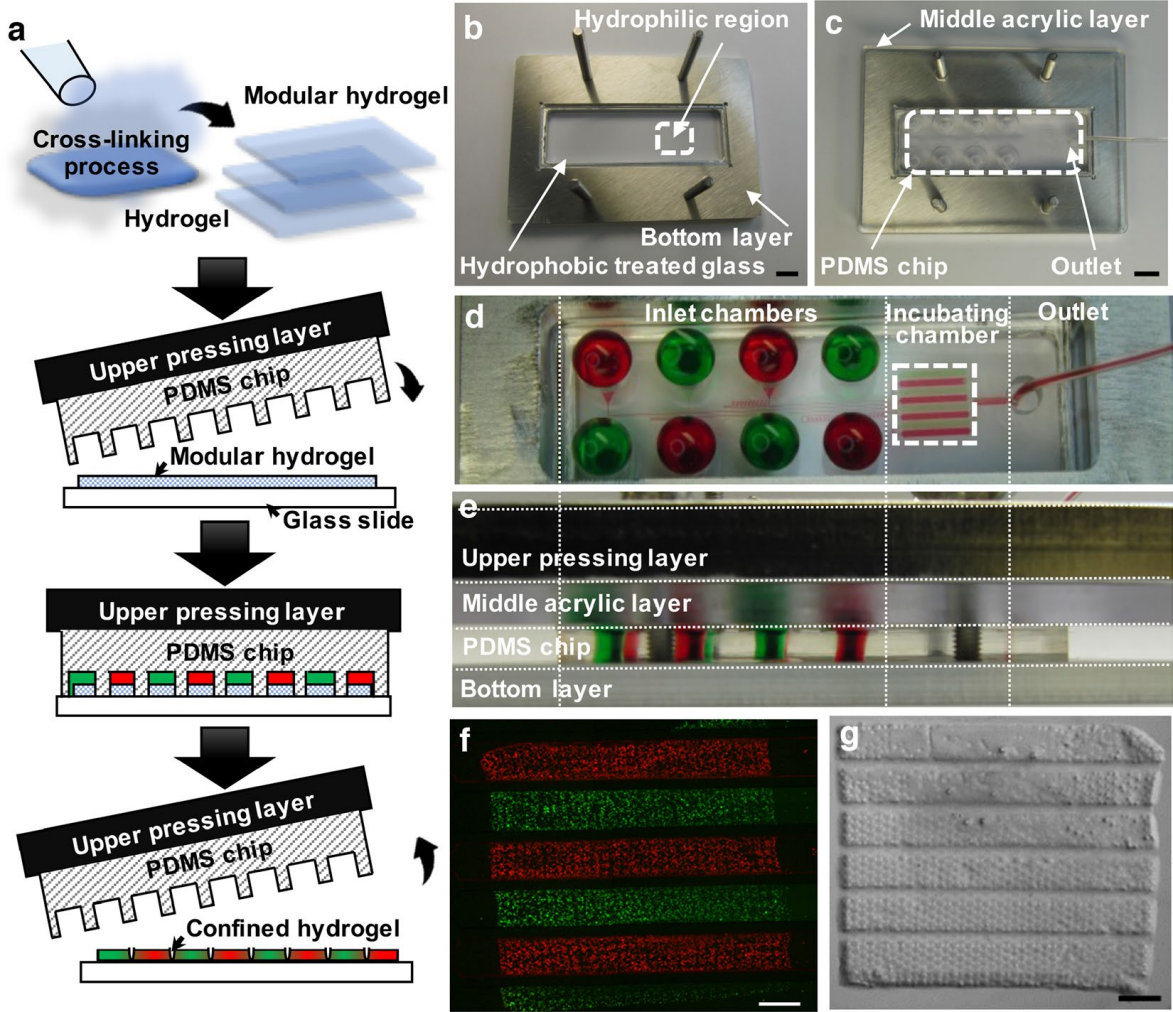
Overview schematic and images of a hydrogel-integrated microfluidic assembly platform. (**a**) Modular hydrogel sheets were massively fabricated and manipulated as a cell-carrying modular unit to incorporate them into a microfluidic device. After microfluidic reactions were performed, the structure of the partially treated modular hydrogel sheet was retained after removal of the microfluidic device. (**b**,**c**) Assembling process for a microfluidic channel layer (PDMS chip), covering the modular hydrogel sheet on the surface of the Teflon-coated glass slide. (**b**) The modular hydrogel sheet was placed onto the hydrophilic region on the CF_4_-coated glass slide. (**c**) The plasma-treated microfluidic channel layer was then covered onto the modular hydrogel sheet. Scale bar = 1 cm. The top (**d**) and side view (**e**) of the whole microfluidic assembly platform, where each liquid has been filled in the inlet chamber to control and withdraw the fluid toward the outlet side. The recovered modular hydrogel sheet was confirmed both from the fluorescent image (**f**) with two different colors (green or red CellTracker) and the bright-field image (**g**) of the pressed modular hydrogel sheet. Scale bar = 1 mm.

Cell-containing hydrogel sheets were fabricated using our previously published techniques^21, 23, 28^. Each fabricated gel was cultured in a multi-wall plate in a floating manner. According to previous results^23^, a honey-comb structure was utilized for each meshed hydrogel sheet to aggregate MIN6 cells into multicellular clusters within each cavity. Based on this single layer of the modular hydrogel sheet, a microfluidic device was assembled to demonstrate rapid, multiple, and modular assays for the multicellular clusters (Fig. 1a). To interpolate a single layer of the hydrogel sheet into the poly(dimethylsiloxane) (PDMS) chip, we needed to fix it or place it on a flat surface. Fortunately, the hydrogel sheet was able to simply transferred onto a commercial glass slide via an amputated pipette tip^21^; however, the water-containing hydrogel easily floated and moved along the smooth hydrophilic surface of the glass. On the other hand, if the hydrogel sheet was chemically bound to the surface of the glass, it would be difficult to detach as a modular unit for further maintenance after each use within the microfluidic device. Therefore, to interpolate the hydrogel sheet into the microfluidic device without any chemical bonding process, we exploited a hydrophobic boundary to control the position on the glass slide (Fig. 1b). Briefly, each glass slide was partially coated with tetrafluoromethane (CF_4_) to make a hydrophobic boundary, except for a region protected with a piece of adhesive tape. After removal of the adhesive tape, a layer of the modular hydrogel sheet could be added via a drop of water to the relatively hydrophilic area, which was indicated as a loading region. Thus, the water-containing modular hydrogel sheet was simply located in the immobilized water drop without any physical attachment process. In this way, we could simply assemble each component, such as the cell-encapsulated hydrogel sheet and the microchannel-embedded PDMS chip, on top of each other for rapid analysis (Fig. 1c). To cover and seal the PDMS chip on the hydrogel sheet, the surface of the PDMS chip was treated with a plasma cleaner to make a hydrophilic surface for the microfluidic channel layer. The hydrophilic surface of the PDMS chip was then placed in contact with the modular hydrogel sheet. This process was carried out to prevent the generation of air bubbles between the hydrogel sheet and the microfluidic channel structure after the assembly of the whole system.

To demonstrate multiple analyses with a single layer of the hydrogel sheet, the PDMS chip was designed with eight isolated microchannels containing eight inlet chambers and eight incubating channels (see Supplementary Fig. S1). The microfluidic channel layer, which was designed as a bottom layer (75 mm × 26 mm) with a commercial glass slide, included eight independent inlet chambers and continuous microfluidic channels with identical flow resistance and a single outlet for drainage. The eight inlet chambers could be filled with culture media or staining solution, depending on the purpose of the experiments (Fig. 1d). After filling the inlet chambers without any bubbles, the flow rate was accurately and constantly controlled by a syringe pump connected to the outlet. Each isolated microchannel combined at the outlet, through which the syringe pump could withdraw the liquid. To properly assemble each layer, a transparent acrylic plate with eight inlet holes and an outlet was incorporated as a middle layer, which allowed a uniform pressure distribution during the assembly process with the PDMS chip (Fig. 1e). Otherwise, the hydrogel could be damaged or torn off due to a non-uniform pressing process, and the flow rate could not be controlled because of an off-balanced microfluidic channel due to an irregularly pressed PDMS chip. Finally, to press the microfluidic channel layer onto the hydrogel sheet, a certain amount of weight was temporarily applied on the microfluidic channel layer as an upper pressing layer. The device could also be screwed and fixed into a frame, so that each component incorporated into the whole microfluidic assembly platform could be transferred from the incubator to the microscope without any instability. Multiple liquid conditions could flow through the separate microfluidic channels of the PDMS chip to interact with spatially segregated regions of the hydrogel sheet. Therefore, only a portion of the total encapsulated cells in the hydrogel sheet was locally exposed to a distinct chemical condition dependent on the type of loaded liquids in the inlet chamber (Fig. 1f). Despite this assembly process, however, the hydrogel sheet was still fully recoverable as a whole structure after the PDMS chip was disassembled (Fig. 1g). When the microfluidic device was removed, the compressed hydrogel sheet could be conserved on the glass slide without any physical distortion or separation. Even though a portion of the modular hydrogel sheet was squashed due to the microchannel wall of the PDMS chip, the entire hydrogel sheet could be harvested and suspended in a floating manner without distortion or tearing apart. To examine the compressed region of the hydrogel sheet, its cross section was validated by bright field microscopy (see Supplementary Fig. S2). The height of the compressed region in the hydrogel sheet (52.41 ± 2.91 µm) was approximately 55.6% of its original height (94.34 ± 2.89 µm). An actual value of the compressed height might be more reduced or compressed than that we have measured, however, its physical features can also be recovered due to an elastic property of the hydrogel material. Thus, a single layer of hydrogel sheet could be used as a modular unit to maintain cells in the *in vivo*-like 3D hydrogel and the cells could be locally stimulated or exposed to multiple chemical conditions through this microfluidic system.

### Leakage confirmation of the microfluidic assembly platform

**Figure 2:**
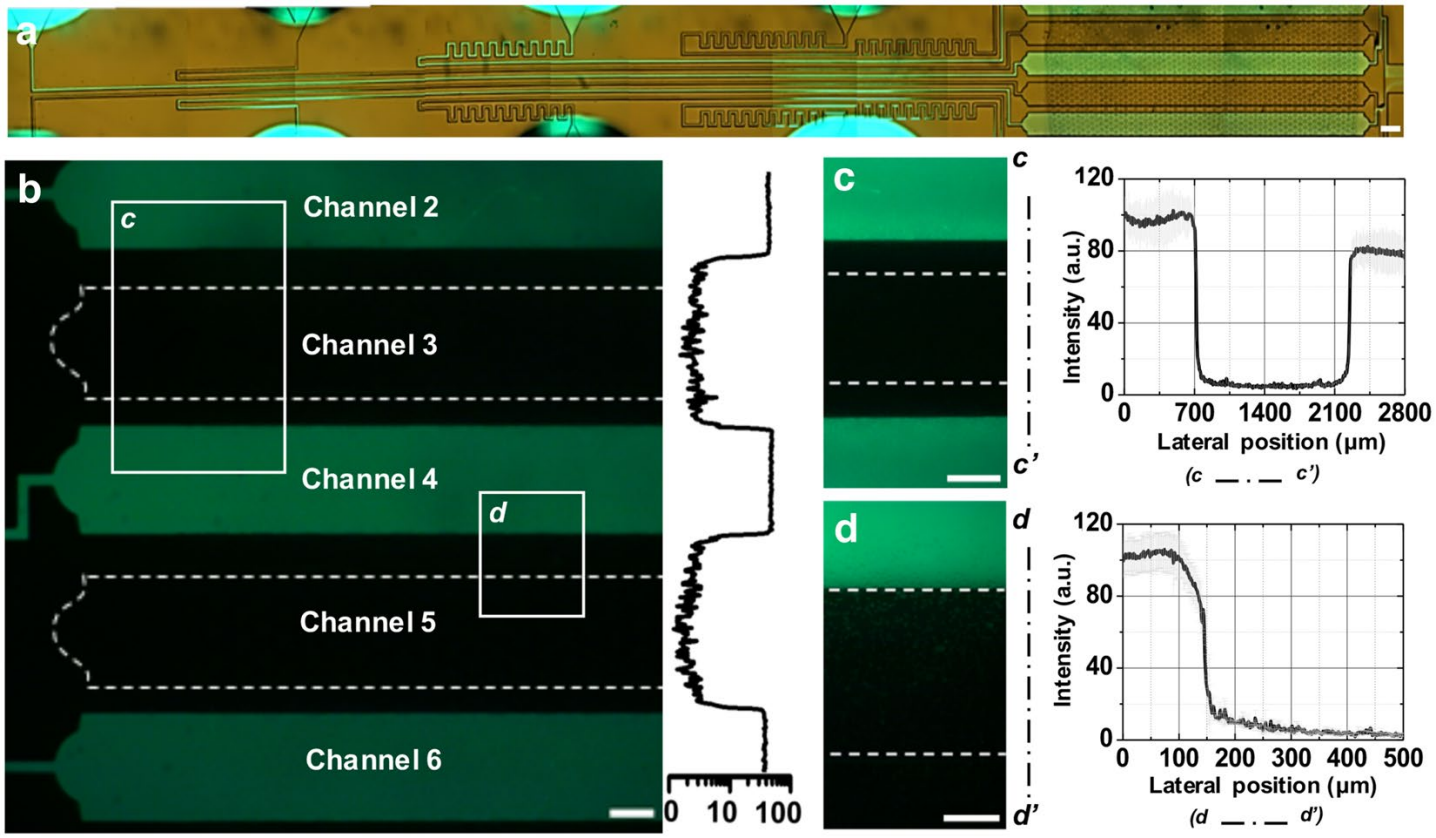
Confirming the sealing between the PDMS chip and the modular hydrogel sheet, including biotin-conjugated polystyrene beads, without any cross-contamination. (**a**) Bright image of the microfluidic assembly platform where the streptavidin–FITC solution passed through only the even-numbered microchannels. (**b**–**d**) The brightness of the encapsulated polystyrene beads and the leaked FITC solution were examined for the sealing effect of the microfluidic assembly platforms in fluorescent images and intensity profiles. (**c**,**d**) Show enlarged images and their intensity profiles as indicated by the boxes in panel b. Scale bar = 500 µm (**b**,**c**) and 100 µm (**d**).

To characterize and confirm the perfectly sealed PDMS chip onto the modular hydrogel sheet, we specifically determined the optimal dimensions of the incubating channels as follows: 200 µm height, 1000 µm width, and 300 µm distance between two adjacent channels (see Supplementary Fig. S1). To demonstrate the optimal design of the PDMS chip, biotin-conjugated polystyrene beads were used; this confirmed that the confined hydrogel sheet was pressed and sealed perfectly (Fig. 2a). To confirm that there was no cross-contamination between the eight incubating chambers, a streptavidin–fluorescein isothiocyanate (FITC) solution was flowed through the even-numbered channels, while a non-fluorescent solution was perfused through the odd-numbered channels (Fig. 2b). According to the intensity profile of the fluorescent images across the channel wall (Fig. 2c and d), there was no leakage between the channels even though portions of the hydrogel were squeezed underneath the PDMS chip. Although some of the encapsulated beads underneath the PDMS channel wall faintly reacted to the FITC solution because of the squeezed hydrogel, no responding beads were found in the adjacent channel. Therefore, the flowing solution was isolated to each incubating chamber and was only able to affect the confined portions of the hydrogel.

**Figure 3:**
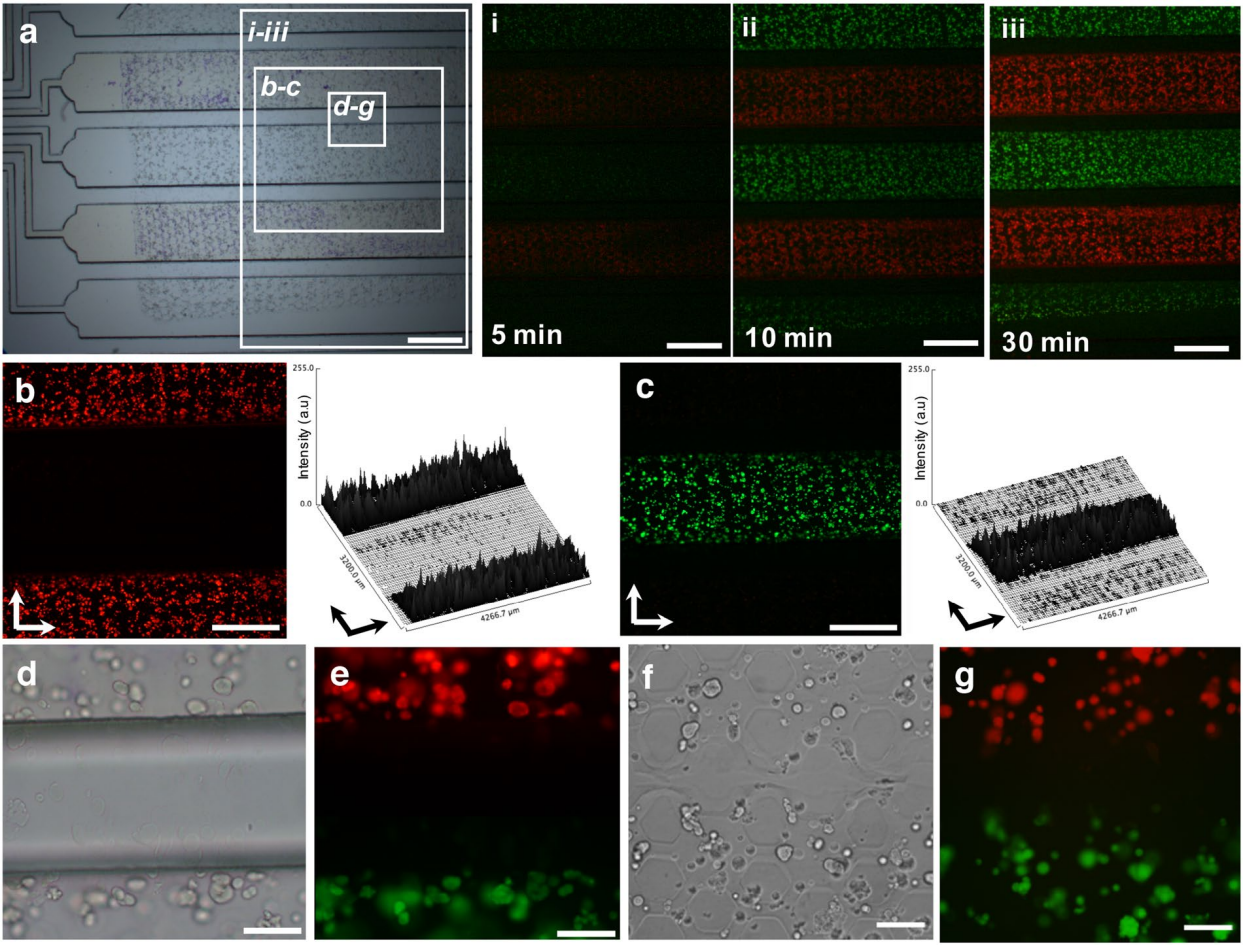
Leakage confirmation of the MIN6 cell-encapsulated modular sheet with two different colored CellTracker (red/green) in the microfluidic assembly platform. According to the incorporating modular sheet (**a**), encapsulated MIN6 cells were steadily stained for 5 min (i), 10 min (ii), and 30 min (iii) with a constant flow rate (40 µL/h) via microfluidic channels. (i–iii) Time-lapse images show as indicated by the box in panel a. (**b**,**c**) The isolated staining of red (**b**) and green (**c**) colored MIN6 cells was assessed for leakage confirmation by fluorescence images and surface plots for their intensity. Scale bar = 1 mm. (**d**–**g**) Two different colors of stained MIN6 cells in the modular sheet were visualized at the border line of the two different channels under the bright field images (**d**,**f**) and the fluorescent images (**e**,**g**), when placed under microfluidic assembly platform (**d**,**e**) or off-microfluidic assembly platform (**f**,**g**). (**b**–**g**) Show enlarged images, as indicated by the boxes in panel a. Scale bar = 100 µm.

To demonstrate cellular analysis with the cell-encapsulated modular sheet in the microfluidic assembly platform, encapsulated MIN6 cells were labeled with a fluorescent dye solution through the confined incubating chamber in the PDMS chip. As shown in Fig. 3a, the fluorescent dye solution was loaded into the incubating chamber, alternating between two different colors (red and green). Controlling a low flow rate in the integrated microfluidic device is important since cells within the 3D hydrogel sheet could be stimulated and damaged under rapid shear stress. Here, we adjusted the total flow rate to be under 40 µL/h for 30 min. Then, under this dynamic condition with flow control, a time-dependent reaction was observed at (i) 5, (ii) 10, and (iii) 30 min after the loading of the staining solution (Fig. 3a, i–iii).

According to the images, the fluorescent intensity of the labeled cells increased as more dye solution passed through the channel. A time-dependent reaction was observed under dynamic condition with flow control. To confirm that there was no leakage between the incubating chambers, a surface profile of the fluorescent intensity was investigated from either red or green colored images (Fig. 3b and c). Both red and green colored images showed that encapsulated cells were only locally affected by fluorescent dyes. Encapsulated cells in the pressed portion of the modular sheet were rarely stained either in color. Although the pressed cells might be damaged (Fig. 3d), it was confirmed that the cells isolated by the incubating chambers were not cross-contaminated with different colored dyes (Fig. 3e). After the PDMS chip was removed, the encapsulated cells in the pressed region from the microchannel wall were not conserved (Fig. 3f), even though the compressed hydrogel sheet still remained intact on the glass slide without rips or separation. Therefore, the whole cell-encapsulated hydrogel sheet was not torn but just successfully compressed. According to Fig. 3g, the cells in the pressed region were unlabeled as a striped mark in the modular hydrogel sheet. Although the width of the channel wall was approximately 300 µm, the actual unlabeled region in the modular hydrogel sheet was smaller than we expected. Thus, the cell-encapsulated hydrogel sheet was successfully applied to the microfluidic assembly platform in order to simultaneously demonstrate multiple reactions under dynamic fluidic conditions.

### Cell viability and proliferation in the modular hydrogel sheet for microfluidic assembly platform

**Figure 4:**
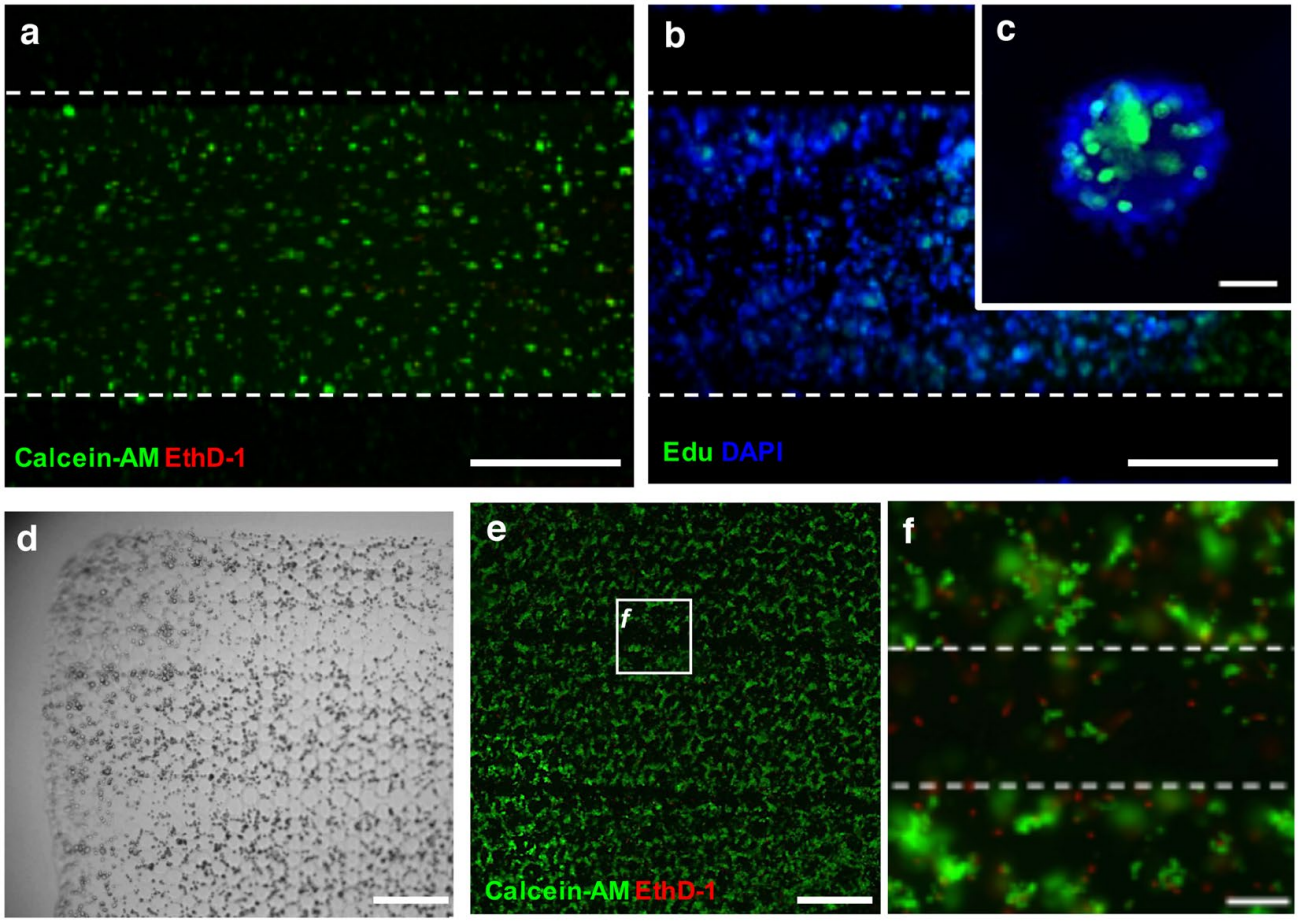
Maintenance of encapsulated cells in the modular sheet inside/outside of the microfluidic assembly platform. Encapsulated MIN6 cells in the modular hydrogel sheet under the microfluidic assembly platform were assessed for their viability (**a**, live (calcein-AM) and dead cells (EthD-1, ethidium homodimer-1) on day 3) and proliferation (**b,c**, replicated nuclei (EdU, 5-ethynyl-2′-deoxyuridine) and nuclei (DAPI) on day 14) under the microfluidic assembly platform for 1 h. (**a**,**b**) The dotted line indicates a boundary of the incubating chamber in the PDMS chip. (**d**–**f**) The pressed modular sheet was obtained (**d**) and the viability of the encapsulated MIN6 cells were also assessed (**e**). Scale bar = 500 µm. (**f**) The dotted line indicates a boundary of the microchannel wall in the PDMS chip. (**f**) Shows an enlarged image, as indicated by the box in panel e. Scale bar = 100 µm.

To confirm the cellular viability and proliferation under the shear-inducing condition in the microfluidic assembly platform, encapsulated MIN6 cells were qualitatively analyzed by staining with calcein-AM (green, live cell) and ethidium homodimer-1 (red, dead cell), or incubating Edu for 2 h (Fig. 4a and b). Due to these staining processes within the microfluidic assembly platform, only a locally exposed region in the hydrogel sheet was examined for viability and proliferation. Particularly, proliferating MIN6 cells in multicellular clusters, generated in the hexagonal meshed hydrogel sheet for 10 days, were verified via labeling bound EdU under the microfluidic assembly platform. According to the magnified image for a certain multicellular cluster (Fig. 4c), only a portion of MIN6 cells within the whole cluster were labeled with green-colored EdU positive among DAPI (blue) stained cells. It was quantitatively determined that approximately 31.6 ± 3.3% among the total cells proliferated in the multicellular clusters from the hexagonal meshed hydrogel sheet. During the incubation of EdU in the microfluidic assembly platform for 2 h, encapsulated MIN6 cells in the modular sheet were confirmed as viable and proliferating under the shear-inducing condition in the microfluidic assembly platform. Through the manipulation of the hydrogel sheet as a module for incorporation into the microfluidic assembly platform, each modular sheet enabled to obtain again and continuously retain as previously assembled (Fig. 4d). A stripe pattern of the squashed hydrogel sheet indicated that these thin structures of hydrogels were sustainable during the process in the microfluidic assembly platform despite an adequate mechanical pressure. Even though a portion of the region, up to 15% of this modular hydrogel sheet, has been squashed down, the modular sheet has been recovered and preserved. Most viable cells have wandered off the hydrogel sheet to make an empty stripe pattern in the retrieved modular sheet (Fig. 4e). In accordance of the enlarged image (Fig. 4f), only small numbers of viable cells remained in the squashed region indicated by two white dotted lines. Consequently, the assembly process has caused the cells to detach from the hydrogel sheet rather than damage them.

**Figure 5:**
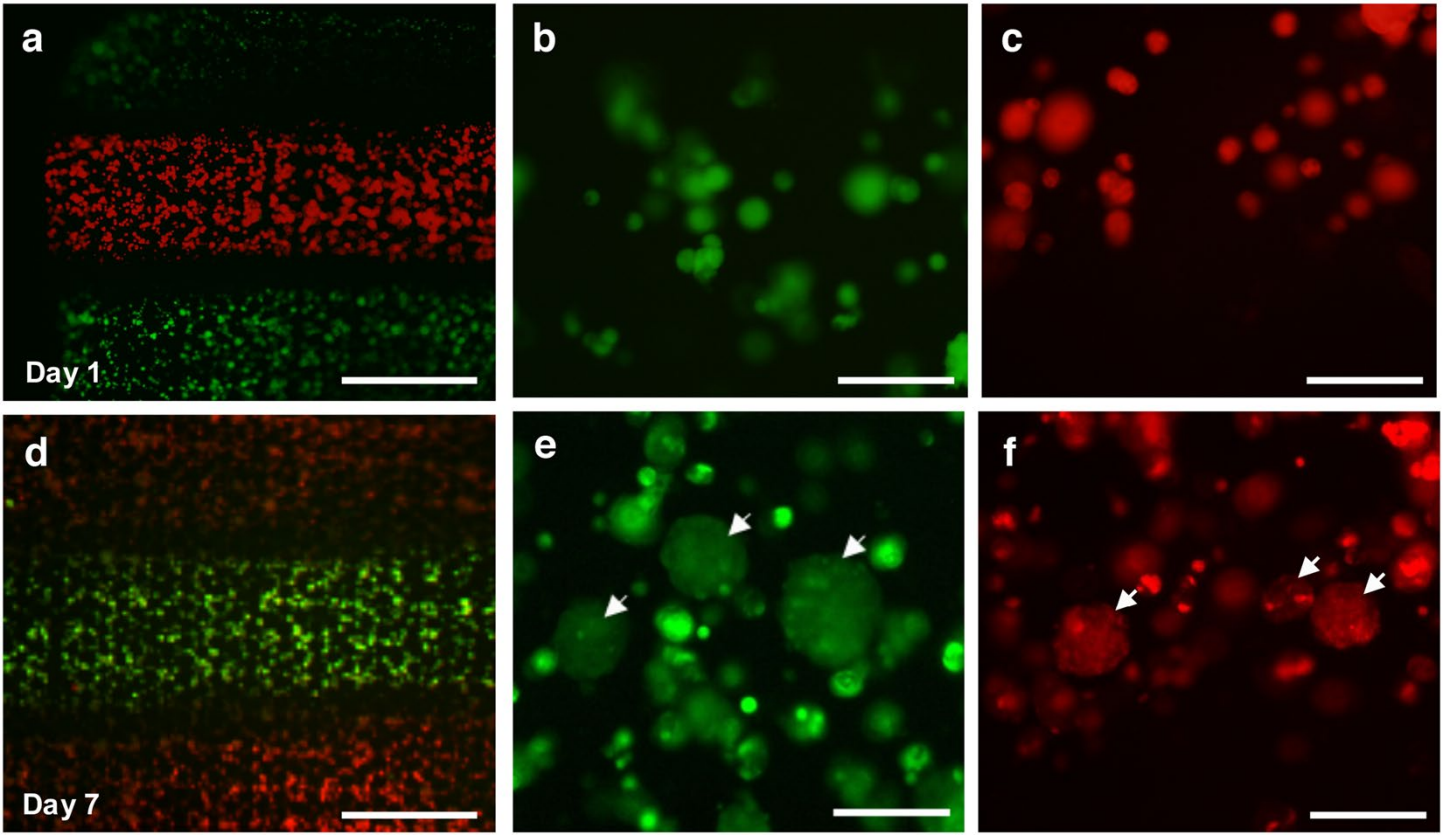
Demonstration of a sustainable modular sheet after the microfluidic assembly platform. (**a**,**d**) Encapsulated MIN6 cells in the harvested modular hydrogel sheet were partially stained and their isolated colors were maintained for an additional 7 days. Scale bar = 1 mm. Single distributed MIN6 cells (**b**,**c**) in the modular sheet were proliferated and cultured for 7 days and then generated into the multicellular clusters (indicated by white arrows) along the micro-sized cavities in the modular sheet (**e**,**f**). Scale bar = 100 µm.

On the basis of checking viability in the modular hydrogel sheet, these compressed modular hydrogel sheets could be stably transferred and floated in the culture medium. After a portion of the hydrogel sheet was stained with two different fluorescent dye solutions, a stripe pattern was obtained on the modular hydrogel sheet which had been incorporated into the microfluidic device (Fig. 5a). The reobtained hydrogel sheet represents the stained MIN6 cells depending on the regions which have just been exposed to green and red dyes. According to the enlarged images of both green and red stained MIN6 cells on day 1 (Fig. 5b and c), MIN6 cells were randomly distributed in the hydrogel sheet and strongly stained with two different colors. After 7 days of further maintenance in its previous condition in a floating manner, both red- and green-colored-stained MIN6 cells were investigated in the compressed modular hydrogel sheet (Fig. 5d). Then, these hydrogel sheets have generated multicellular clusters at the cavity of the meshed structure in a static culture as they used to do (see Supplementary Fig. S3). As time passed by, an amount of dye solution in each cytosol was divided and decreased when the cells were successfully replicated. On the basis of the decreased intensity of fluorescence in those multicellular clusters at day 7, it was assumed that the stained single MIN6 cells remained viable under the microfluidic device and replicated after the assay (Fig. 5e and f). Consequently, procedures of the modular assay in a microfluidic device can be totally separated from cell culture and long-term maintenance in the hydrogel scaffold. Furthermore, after assay procedure, each modular sheet was recoverable and was used to re-culture as a culture module in a floating manner. Therefore, the modular hydrogel sheet in the microfluidic assembly platform can be demonstrated as a 3D cellular unit model, and showed a potential application for further validation after the integrated analysis process.

### Multiplexed, rapid analysis of 3D modular hydrogel sheet in a microfluidic assembly platform

**Figure 6:**
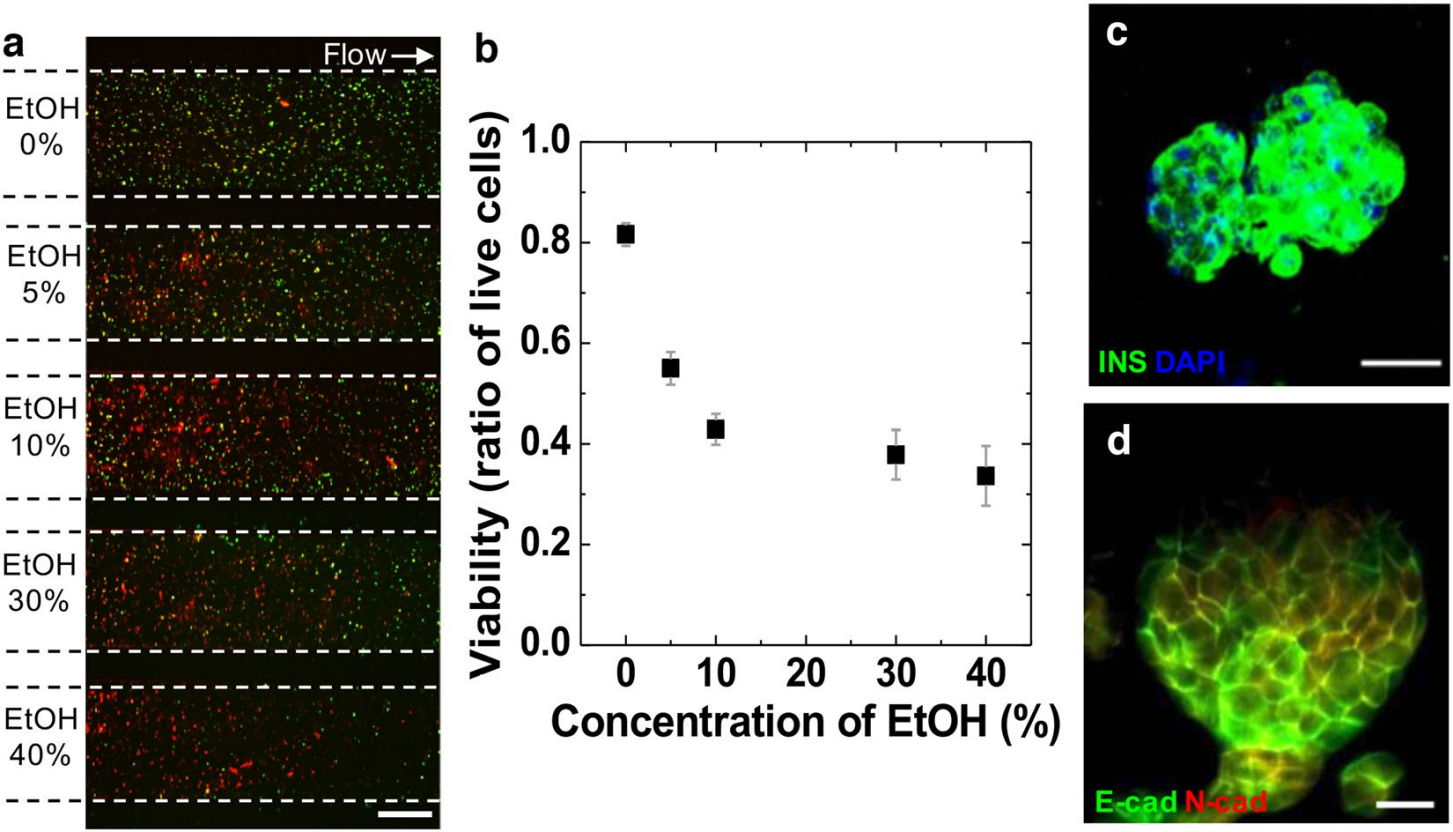
Demonstration of the assessment of the chemical response in the modular hydrogel sheet encapsulating MIN6 cells. (**a**,**b**) The cell viability was qualitatively or quantitatively examined depending on the various concentrations (0%, 5%, 10%, 30%, and 40%) of ethanol (EtOH) via multiple incubating chambers in the microfluidic assembly platform for 1 h. Scale bar = 500 µm. (**c**,**d**) Multicellular clustered MIN6 cells in the modular sheet on day 14 were assessed by flow-based immunostaining for insulin (INS) (**c**, green), E-cadherin (E-cad) (**d**, green), and N-cadherin (N-cad) (**d**, red) in the microfluidic assembly platform. Scale bar = 20 µm.

To simply demonstrate multiple analyses of the incorporating modular hydrogel sheet into the microfluidic device, encapsulated MIN6 cells were validated at once at a certain chemical response with several concentrations in the multiple microchannels (Fig. 6a). A generated hydrogel sheet, encapsulating MIN6 cells, was integrated with the microfluidic channel layer. Ethanol (EtOH) was then treated as a model chemical for 2 h at five different concentrations. When the medium was treated (0%), the viability was over 80%. As the concentration of EtOH increased, on the other hand, the viability of the encapsulated MIN6 cells in the single modular hydrogel sheet decreased. According to the five-separated microchannels with different conditions of EtOH, a certain area for each channel among the single hydrogel sheet was implemented with different aspects of viability, particularly in the dead cell number, depending on the chemical conditions being treated. The quantitative analysis of the viability of the encapsulated MIN6 cells in the modular hydrogel sheet was also determined based on the live/dead stained images (Fig. 6b). It was clear that the viability was diminished in the microchannel containing a high concentration of EtOH. Therefore, the viability of the cells within the single modular hydrogel sheet was clearly confined into the five different sections via microchannels through which five different conditions of the liquid solution were infused. Then, multiple liquid conditions were analyzed in the single modular hydrogel sheet using a microfluidic integrated device.

The localization of certain proteins was also validated by immunostaining in the generated MIN6 multicellular clusters within the modular hydrogel sheet, incorporated in the microfluidic integrated system. Without the flow-driven process in the microfluidic integrated system, the incubation of the primary antibody and the permeation of cells can be treated statically overnight. After the fixation of the modular hydrogel sheet, on the other hand, the MIN6 cells could be labeled with insulin, E-cadherin and N-cadherin within 2 h in the integrated microfluidic device at a flow rate of 40 µL/h. Insulin was localized in the cytoplasm of each MIN6 cell (Fig. 6c), and both N-cadherin and E-cadherin were stained following cell boundaries, with adjacent cells interacting with each other (Fig. 6d). In particular, E-cadherin was increased at the centered clusters, with the cells being relatively more strongly attached than the interaction at the boundary, as multicellular clusters were growing (see Supplementary Fig. S4). Furthermore, the expression of N-cadherin in the earlier state of MIN6 multicellular clusters was higher due to less adhesion and more migration between the cells.

## Discussion

We have reversibly integrated a layer of hydrogel sheet as an insertional module into a microfluidic device, which aims to demonstrate the potential application of a hydrogel module-based multicellular analysis. To present an optimum process for incorporating the hydrogel sheet, a universally operated microfluidic interface was proposed based on an independent hydrogel sheet. When optimizing this microfluidic assembly platform, primary design factors must be considered. First of all, the height of the microchannel would be one of the important design factors for the conserved hydrogel sheet as interpolation. When the upper pressing layer was placed onto the top of the whole platform, the incorporating hydrogel sheet would be squeezed by an elastically deformable PDMS chip, which also contained compressible microfluidic channels with approximately 200 µm in height. If they were not high enough to cover the hydrogel sheet, it would be impossible to run each solution via microfluidic channels due to a blockage of the hydrogel in the incubating chamber (see Supplementary Fig. S5). As a result of the examination of various heights of the microfluidic channel layer, it was at least appropriate to suggest more than 200 µm in height of the microfluidic channel to cover the 100 µm-modular sheet. Next, a gap size between the channel walls was the most significant factor against the leakage of the solution in the PDMS chip. To investigate cross-contamination inside the pressed hydrogel sheet, a streptavidin–FITC solution was introduced to the microfluidic assembly platform based on the modular hydrogel sheet, encapsulating biotin-conjugated 10-µm-polystyrene beads instead of cells. In the case of a 200 µm gap between the channel walls, embedded biotin-conjugated beads beneath the channel wall were brightly responded to the streptavidin–FITC solution due to the interaction between encapsulated biotin-conjugated beads and leaked streptavidin–FITC solution (see Supplementary Fig. S5). Based on this demonstration, we estimated that it is possible for encapsulated cells to be affected and/or contaminated by the leaked solution at a distance of less 200 µm between the walls. In compliance with optimized dimensions in the microfluidic channel layer, we minimized a region of the hydrogel pressed by the PDMS chip.

MIN6 cells, which we demonstrated in this hydrogel sheet incorporated in the microfluidic assembly platform, can be a useful substitute for pancreatic primary beta cells that needed to be clearly understood for diabetes and its therapeutics. Although beta cell failure is related to a progress of diabetes, however, there was not much way to determine beta cell failure during a drug screening procedure, especially in an *in vitro* microenvironment, because it is difficult to maintain its viability *in vitro*. To screen several drugs related to diabetes under the multicellular structures of MIN6 cells as a substitute for pancreatic beta cells, it would therefore be necessary to understand their viability, toxicity and even proliferation as comparable primary beta cells. In order to perform a biological in-depth drug screening research using this microfluidic assembly platform, there are still a few more things to be considered. MIN6 multicellular structures should be fully understood in this hydrogel construct during the culture period, because they might have different aspects of the biological function compared to primary beta cells. Beta cell specific drugs can also be treated for the toxicity test of MIN6 cells in these hydrogel sheet. For example, in this microfluidic assembly platform, when we tested streptozotocin (STZ), a naturally occurring chemical compound to produce an animal model for hyperglycemia, it seemed to affect the MIN6 cell viability depending on its concentration (see Supplementary Fig. S6). However, the various drug responses under the hydrogel sheets should be determined in order to characterize a beta cell specific drug screening system using MIN6 multicellular structures. Consequently, we developed the microfluidic assembly platform which can show a possibility to analyze the viability, toxicity and proliferation of multicellular structures in the hydrogel sheet. In order to provide further in-depth biological insights via this microfluidic assembly platform, it is also necessary to investigate biochemical experiments based on multicellular structures in the hydrogel sheet.

One of the significance in this work is that both the culture and the analysis process can be separated and proceeded. Previous methods have focused more on performing to generate those spheroidal shapes of multicellular structures rapidly and effectively based on the specific substrates^2, 11, 15^ or microfluidic droplet techniques^10^. However, it has been restricted to apply them to the microfluidic device for certain reasons; for example, thousands of each multicellular structure in the microfluidic devices should be adjusted, manipulated, and rearranged. Moreover, due to the rapid generation of multicellular structures, cells reproducing themselves may exhibit various aspects of problems such as necrotic regions at the core of the spheroids, and over growth at the status of spheroids. On the other hand, the hydrogel sheets can support (1) multiscale micropatterning of the scaffold for self-clustered spheroids or biomimetic geometry, (2) simple transferable layers with macroscale surface, (3) reorganizing as a multicellular structure in a modular scaffold, and (4) tracking encapsulated cells within the hydrogel sheet. Thus, the microfluidic assembly platform suggested the various applications and their procedure for analyzing multicellular structures based on the integration of the modular hydrogel sheet. According to our previous study^23^, the hydrogel sheet itself can provide the inner meshed constructs for aggregation into multicellular clusters. Without losing its own original effect, this hydrogel sheet was widely adopted to the microfluidic assembly platform. Meanwhile, sheet-like hydrogel constructs were applied only to a simple, effective manipulation of rearranged multicellular structures in a single layer^21, 23^, or a novel application for enlarged tissue-like multiple layers^22, 24, 25^. However, there were no reports on the analysis of multicellular structures on a large contact surface of the hydrogel sheet in a dynamic fluidic condition under the microfluidic device. This is the first report on the incorporation of a hydrogel sheet, having a large-surface-area, into the microfluidic assembly platform. Based on this demonstration, we specifically expect that this system to be applicable to multi-dose multi-drug treatment in a single hydrogel sheet and the analysis of drug effects based on the long-term proliferation of multicellular clusters. Therefore, the proposed system could be applicable to a 3D multicellular assay with dynamic flow control and to a multiplexed rapid screening using a 3D modular hydrogel sheet.

## Methods

### Fabrication and manipulation of a hydrogel sheet

To generate and analyze multicellular clusters, single distributed MIN6 cells were encapsulated in a hydrogel sheet, including a honey-comb microstructure^23^. The MIN6 cell-encapsulated hydrogel sheet was incubated with the MIN6 cell-specific medium (Dulbecco’s modified Eagle’s medium with 4.5 g/L glucose supplemented with 15% fetal bovine serum, 100 mg/L penicillin–streptomycin and 71.5 µM 2-mercaptoethanol). The hydrogel sheet was simply and massively generated using 2%(w/v) sodium alginate via a previous molding technique^21, 23, 26^. To accurately fabricate a 100 µm-thick-structure of the hydrogel sheet, a cross-linking reagent (100 mM, CaCl_2_·2H_2_O was treated in a manner of the mist using a humidifier. Once a certain shape of the hydrogel sheet has been formed, then it is also easy to dissolve it under the phosphate buffered saline (PBS) without any calcium ions. Therefore, it could be transferred or manipulated in a buffered solution or a washing solution based on the Dulbecco’s PBS with calcium and magnesium (DPBS; Invitrogen).

### Components of a microfluidic assembly platform

A PDMS chip for a microfluidic assembly platform was designed in consideration of the whole size of the modular hydrogel sheet (8 mm × 8 mm × 100 µm), particularly from the height of the hydrogel sheet. Based on its physical condition, the height (approximately 200 µm) of each microfluidic channel in the PDMS chip was constructed to fully cover a height of the hydrogel sheet. The length of each microchannel between the inlet chambers and incubating channels was designed for the identical fluidic resistance. One single outlet was connected to the syringe pump (PHD 2000; Harvard Apparatus, Holliston, MA, USA), which withdrew the whole fluids from the eight independent inlet chambers. The mold of the PDMS chip for the microfluidic assembly platform was fabricated via conventional soft lithography, as previously reported^29^. To construct a microfluidic device, SU-8 2100 (MicroChem Corp., Westborough, MA, USA) was spin-coated to form a 200 µm-height on a bare silicon wafer, patterned by ultraviolet (UV) exposure. After pouring and curing the mixture of PDMS (Sylgard Silicone Elastomer; Dow Corning, Auburn, MI, USA) and curing agent (Sylgard Curing Agent 184; Dow Corning) onto the mold of the microfluidic channel layer, both an outlet and inlets of each PDMS chip would be punched.

Similar to our previous integrated system multiplexed immunohistochemistry for cancer diagnosis^29^, the microfluidic assembly platform for the modular cell-encapsulated hydrogel sheet was also organized into three different parts, such as a bottom layer, a microfluidic channel layer and an upper pressing layer. Both the bottom and upper layers were a custom-designed aluminum holder that is compatible with a commercial glass slide. An adhesive tape (8 mm × 8 mm) was attached to the region of the incubating channel on the glass slide. It was coated with CF_4_, as a polymerizing monomer, to make a hydrophobic boundary using argon atmospheric plasma (IHP-1000; APP Co., Hwaseong, Korea). Then, the glass slide was sterilized after removal of the self-adhesive tape. The modular hydrogel sheet was transferred and simply spread to the hydrophilic region where the self-adhesive tape was blocked. After the hydrogel sheet was placed on the glass slide, the PDMS chip treated by a low-pressure plasma system (CUTE; Femto Science, Yongin, Korea) was covered onto the modular hydrogel sheet. To cover and assemble the PDMS chip on the modular hydrogel sheet without any fluidic leakage, a sealing process was performed using a weight (e.g., 374.3 g, 10 mm thickness) for the integrated microfluidic system. To avoid a possible small dislocation of the PDMS chip, four screws can be used optionally.

### Analysis of multicellular clusters in the modular hydrogel sheet

The encapsulated cells were labeled with a green or red dye (10 µM diluted CellTracker; Invitrogen) for 30 min under the microfluidic assembly platform. To investigate cell replication inside the modular hydrogel sheet under this platform, proliferating cells were also validated by EdU staining (Click-iT EdU Imaging Kits; Invitrogen). An immunofluorescence staining process was also performed on the modular hydrogel sheet, including fixed MIN6 cells treated in 4% paraformaldehyde (16% formaldehyde diluted; Thermo Fisher Scientific) for 10 min. After 30 min treatment of blocking solution (3% bovine serum albumin; Sigma-Aldrich) and 30 min treatment of permeating solution (0.1% Triton X-100; Invitrogen) process, both primary and secondary antibodies with fluorescent tagging were flowing serially at a flow rate of 40 µL/h for 1 h via microchannels. Detailed information about each antibody is as follows; anti-insulin (guinea pig, 1:1000; Dako), anti-N-cadherin (mouse, 1:100, BD Biosciences), anti-E-cadherin (rabbit, 1:100, Cell signaling), FITC-conjugated goat anti-guinea pig IgG (1:1000, Jackson ImmunoResearch Laboratories, Inc., West Grove, PA, USA), rhodamine-conjugated goat anti-rabbit IgG (1:1000, Jackson ImmunoResearch Laboratories, Inc.) and Alexa647-conjugated goat anti-mouse IgG (1:1000, Jackson ImmunoResearch Laboratories, Inc.). 4′,6-diamidino-2-phenylindole (DAPI; Thermo Fisher Scientific) diluted 1:1000 in DPBS was used for nucleic acid staining to assess the morphology of the gross cells.

## Electronic supplementary material

Supplementary Figures S1–S6
