# Supplementary material for: Demonstration of Interposed Modular Hydrogel Sheet for Multicellular Analysis in a Microfluidic Assembly Platform
Source: Sci Rep. 2017 May 2;7:1289. doi: 10.1038/s41598-017-01363-6 (PMC5430983; doi:10.1038/s41598-017-01363-6)
Supplement: Supplementary file 1 — Supplementary Figures S1–S6 [file 41598_2017_1363_MOESM1_ESM.pdf]

Demonstration of Interposed Modular Hydrogel Sheet for Multicellular Analysis in a Microfluidic Assembly Platform

Chae Yun Bae,[1] Jaejung Son,[1] Hail Kim[2] and Je-Kyun Park[1,*]

[1] Department of Bio and Brain Engineering, Korea Advanced Institute of Science and Technology (KAIST), 291 Daehak-ro, Yuseong-gu, Daejeon 34141, Republic of Korea.

[2] Graduate School of Medical Science and Engineering, Korea Advanced Institute of Science and Technology (KAIST), 291 Daehak-ro, Yuseong-gu, Daejeon 34141, Republic of Korea.

*Correspondence and requests for materials should be addressed to J.-K.P. (email: jekyun@kaist.ac.kr)

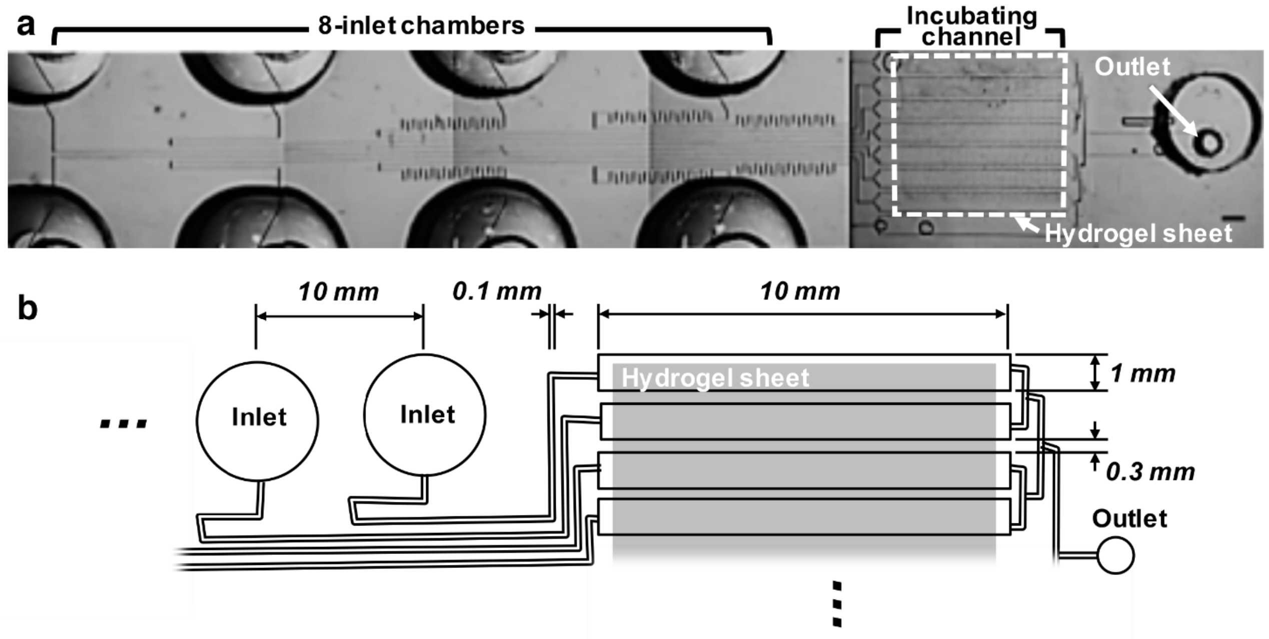

Supplementary Figure S1. Geometry of the microfluidic channel layer. (a) The microfluidic channel layer has eight independent inlet chambers, eight separated incubating channels for covering the modular sheet, and one single outlet for withdrawing the liquid. Each microchannel between the inlet chambers and the incubating channels has the identical length and width for the same resistance. Scale bar = 1 mm. (b) Dimensions of the microfluidic channel layer (height: 0.2 mm). One of the most critical values for this microfluidic channel layer is a gap (0.3 mm) between two adjacent incubating channels (10 mm × 1 mm).

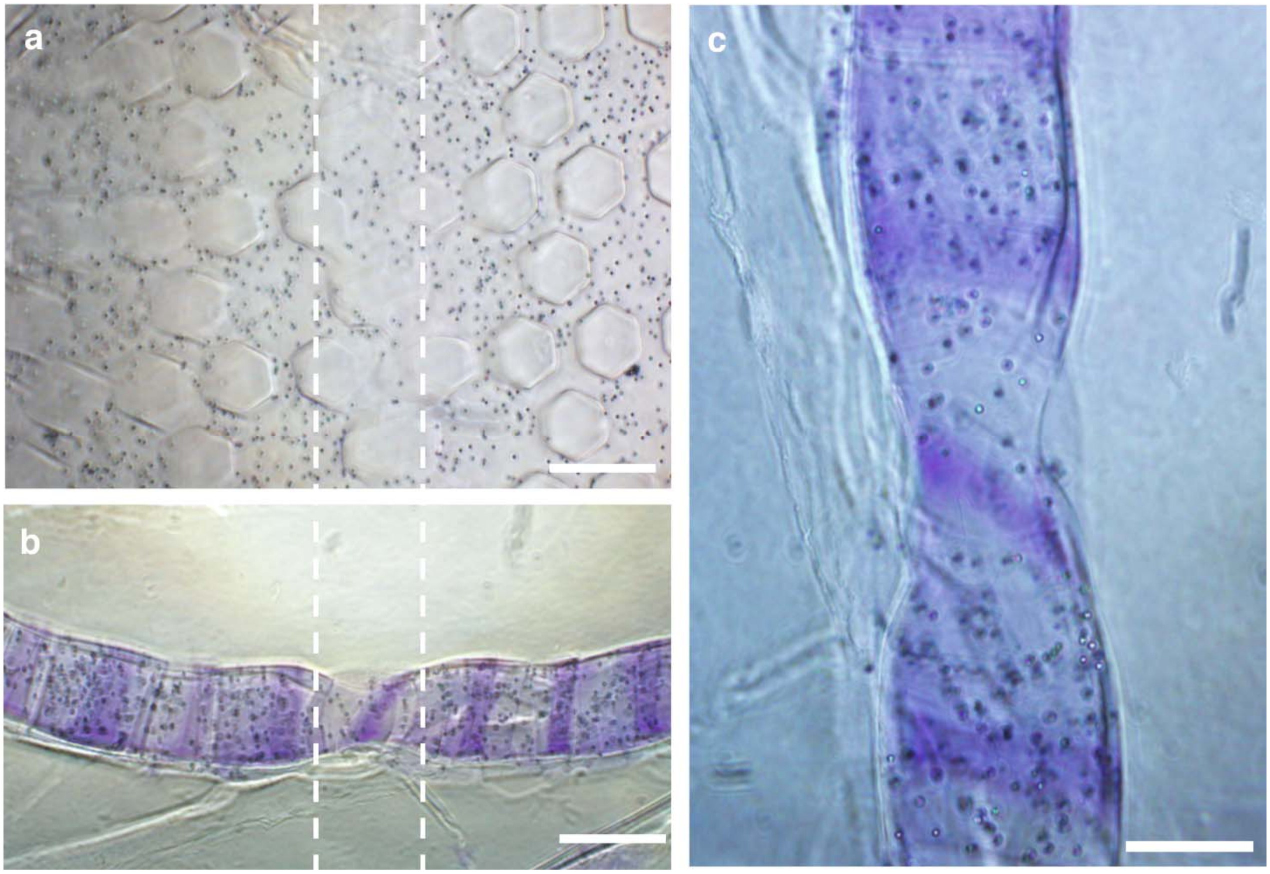

Supplementary Figure S2. Top view and cross section of the compressed hydrogel sheet. (a) Top view of the bead (6 μm polystyrene)-embedded hydrogel sheet. (b) Cross section of the same hydrogel sheet embedded in 1% agarose. Purple color indicates hydrogel scaffolds stained by methylene blue. White dashed line indicates the compressed region of the hydrogel sheet. Scale bar = 100 μm. (c) An enlarged image of the cross-sectional view of the compressed region in this hydrogel sheet. Scale bar = 50 μm.

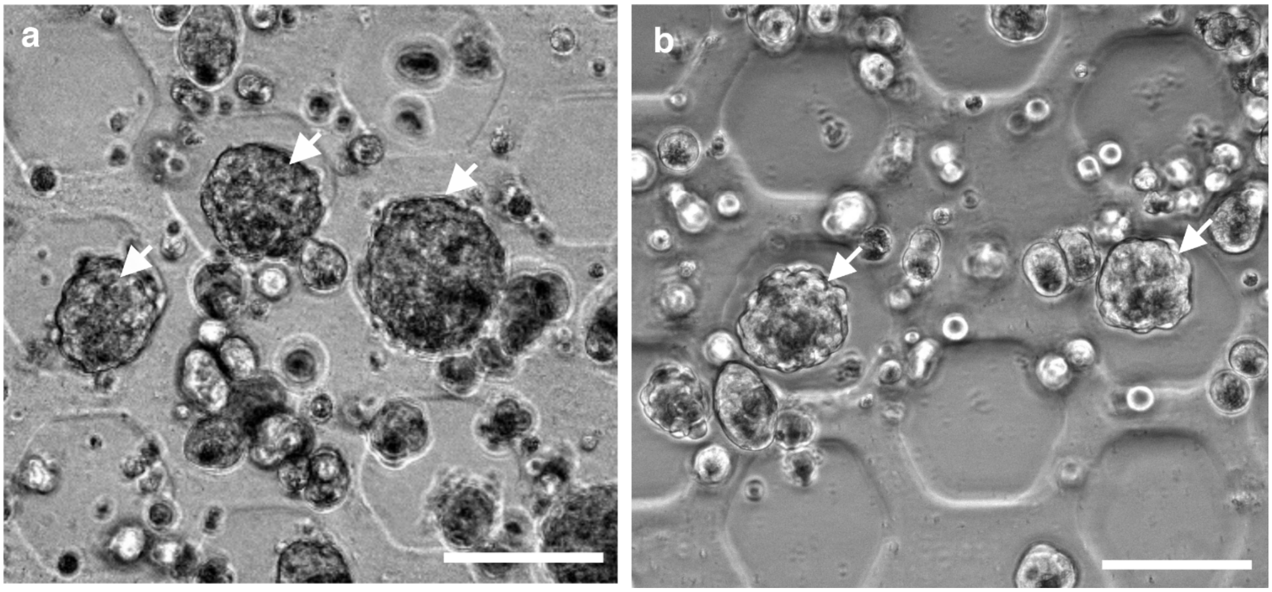

Supplementary Figure S3. Bright field images of generated multicellular clusters in the hydrogel sheet on 7 days after staining procedures under the microfluidic assembly platform. Both green (a, identical image with Fig. 5e) and red (b, identical image with Fig. 5f) were stained in the microfluidic assembly platform and then generated into the multicellular clusters (indicated by white arrows) along the micro-sized cavities in the modular sheet. Scale bar = 100 μm.

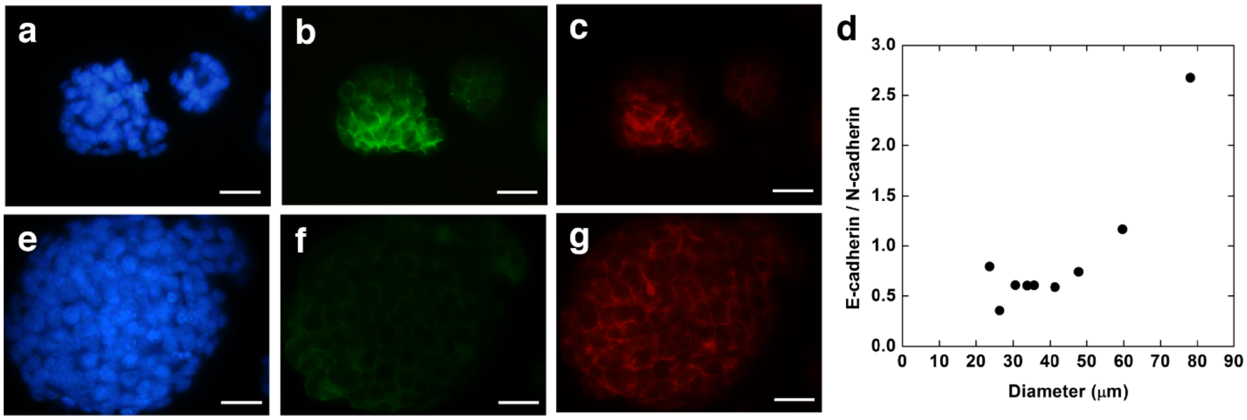

Supplementary Figure S4. E-cadherin and N-cadherin expression of MIN6 multicellular clusters. Two different sizes of MIN6 multicellular clusters were investigated with DAPI (a, d), N-cadherin (b, e) and E-cadherin (c, f). Scale bar = 20 μm. (d) As MIN6 multicellular clusters were growing, a ratio of cadherin (E-cadherin/N-cadherin) also increased because E-cadherin increased.

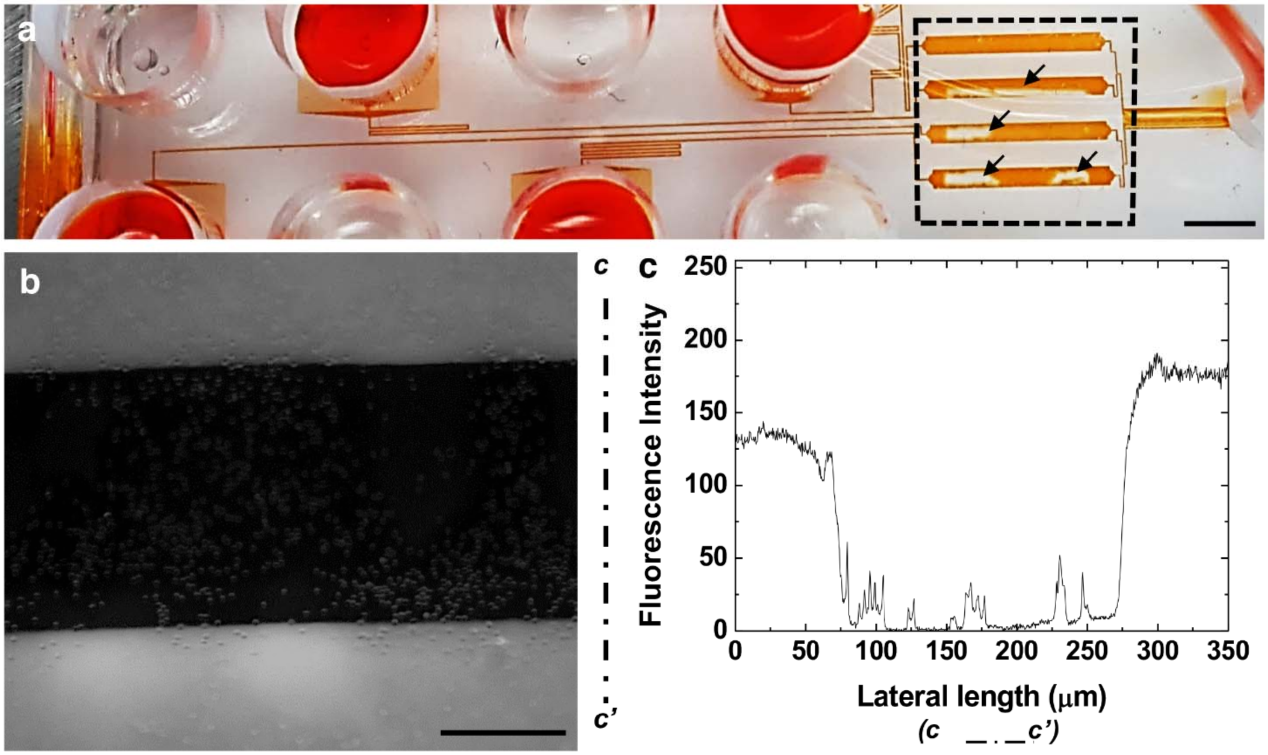

Supplementary Figure S5. Possible troubles in a microfluidic assembly platform. (A) Due to the lower height (150 μm) of the microfluidic channel in a PDMS chip, blockage of the incubating channel (black dotted box) could occur. Then, the pressed hydrogel sheet (black arrow) would be blocked off the passage for the solution. Scale bar = 500 μm. (B) Leakage under the microchannel wall (black region) could be assessed by brightly responded polystyrene beads between the two adjacent incubating channels (white region) due to the narrow gap (200 μm). (C) The fluorescence intensity of the lateral plane between the two adjacent incubating channels indicates that encapsulated biotin-conjugated polystyrene beads were brightly responded to the leaked streptavidin–FITC solution from the microchannel. Scale bar = 100 μm.

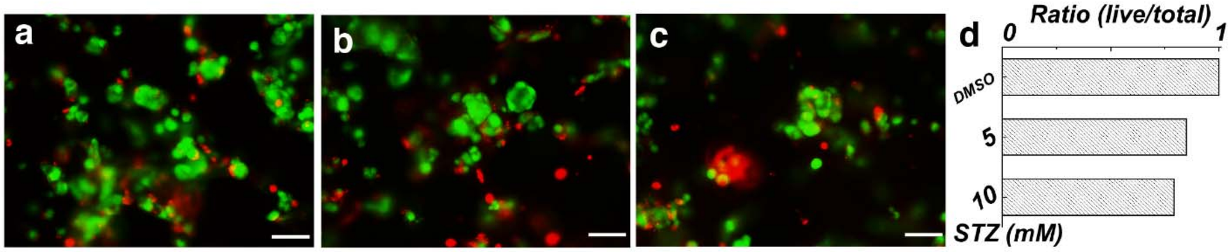

Supplementary Figure S6. Treatment of streptozotocin (STZ) in the modular hydrogel sheet which incorporated in the microfluidic assembly platform. The viability of 1-week-cultured MIN6 cells in the hydrogel sheet was determined depending on different concentrations of STZ such as (a) 0 mM, (b) 5 mM and (c) 10 mM. (d) The ratio of live cells was also quantitatively assessed. Scale bar = 100 μm.
